# Photobiomodulation Modulates Temporal VEGF Dynamics and Tissue Repair During Diabetic Oral Mucosal Wound Healing in Rats

**DOI:** 10.3390/ijms27219395

**Published:** 2026-10-22

**Authors:** Nadica S. Đorđević, Ilija M. Dragojević, Milica Mijović, Jelena T. Todić, Dragan Marjanović, Aleksandar M. Đorđević, Zvezdan Milanović, Julijana Rašić, Bojana Kisić, Zlatibor Anđelković, Miljana Z. Stašević, Milena M. Kostić

**Affiliations:** 1 Department of Dentistry, Faculty of Medicine, University of Priština in Kosovska Mitrovica, 38220 Kosovska Mitrovica, Serbia; nadica.djordjevic@med.pr.ac.rs (N.S.Đ.); jelena.todic@med.pr.ac.rs (J.T.T.); drmarjanovicdragan@gmail.com (D.M.); aleksandar.djordjevic@med.pr.ac.rs (A.M.Đ.); 2 Institute of Biochemistry, Faculty of Medicine, University of Priština in Kosovska Mitrovica, 38220 Kosovska Mitrovica, Serbia; bojanabk2002@yahoo.com; 3 Institute of Pathology, Faculty of Medicine, University of Priština in Kosovska Mitrovica, 38220 Kosovska Mitrovica, Serbia; milica.mijovic@med.pr.ac.rs; 4 Institute of Physiology, Faculty of Medicine, University of Priština in Kosovska Mitrovica, 38220 Kosovska Mitrovica, Serbia; zvezdanmilanovic@yahoo.com; 5 Institute of Pharmacology, Faculty of Medicine, University of Priština in Kosovska Mitrovica, 38220 Kosovska Mitrovica, Serbia; julijana.rasic@med.pr.ac.rs; 6 Institute of Histology, Faculty of Medicine, University of Priština in Kosovska Mitrovica, 38220 Kosovska Mitrovica, Serbia; zlatibor.andjelkovic@med.pr.ac.rs; 7 Faculty of Medicine, University of Priština in Kosovska Mitrovica, 38220 Kosovska Mitrovica, Serbia; miljana.stasevic@gmail.com; 8 Clinic for Dental Medicine, Faculty of Medicine, University of Niš, 18000 Niš, Serbia; milena.kostic@medfak.ni.ac.rs

**Keywords:** photobiomodulation, low-level laser therapy, vascular endothelial growth factor, diabetes mellitus, oral mucosa, wound healing, angiogenesis, tissue repair

## Abstract

Photobiomodulation (PBM) may improve diabetic wound repair, but the temporal relationship between local angiogenic signaling and tissue-level healing remains incompletely defined. We examined previously unreported tissue vascular endothelial growth factor (VEGF) and histopathological outcomes from 72 ulcer-bearing male Wistar rats from a previously reported experimental cohort, using a balanced 2 × 2 × 3 design comprising metabolic status, PBM treatment, and healing day (3, 7, or 10; n = 6 per cell). Tissue VEGF was quantified by ELISA and normalized to total protein; histopathological outcomes included exploratory inflammation/injury and tissue repair composite scores. In the factorial log-VEGF model, diabetes × day (*p* = 0.0017) and LLLT × day (*p* = 0.0010) interactions demonstrated phase-dependent responses. On day 3, PBM-treated animals had an adjusted VEGF ratio of 2.13 versus untreated animals (95% CI 1.63–2.78; Holm-adjusted *p* < 0.001), whereas diabetic animals had lower VEGF than healthy animals (ratio 0.50, 95% CI 0.39–0.66; adjusted *p* < 0.001). VEGF concentrations converged by day 10. PBM remained associated with a higher exploratory tissue repair score after adjustment for healing day, diabetes status, and contemporaneous VEGF, whereas VEGF itself was not independently associated with repair. These findings demonstrate phase-dependent differences in tissue VEGF and suggest that contemporaneous VEGF concentrations do not fully explain treatment-associated variation in exploratory histopathological repair scores.

## 1. Introduction

Oral mucosal repair is distinguished by rapid re-epithelialization and relatively limited fibrosis, yet this efficiency depends on precisely timed interactions between inflammatory cells, keratinocytes, fibroblasts, endothelial cells, and the extracellular matrix. Angiogenic signaling is particularly important during the transition toward proliferative repair, when restoration of a functional microvascular network supports granulation tissue formation and tissue reconstruction. Among the mediators involved, vascular endothelial growth factor (VEGF) promotes endothelial activation, migration, vascular permeability, and neovascular responses, making its temporal regulation especially relevant to the evolving wound environment [1,2,3].

Diabetes alters this temporal coordination rather than simply slowing each component of repair to the same extent. Hyperglycemia can impair endothelial responsiveness, disturb stromal-cell function, prolong inflammatory activity, and interfere with hypoxia-sensitive angiogenic signaling [4]. These effects are particularly relevant in oral mucosa, where normally rapid epithelial turnover, salivary mediators, and a specialized microbial environment create a repair phenotype that differs from that of skin [1,5]. Evidence from diabetic oral wounds further suggests reduced local VEGF responsiveness [6], raising the possibility that diabetes changes not only the magnitude but also the timing of the angiogenic response during healing.

Photobiomodulation (PBM), historically termed low-level laser therapy (LLLT), is increasingly viewed as a context-dependent regulator of cellular signaling rather than simply as a stimulus for wound closure. Light-induced changes in mitochondrial and redox activity can propagate through multiple signaling networks that regulate endothelial function, fibroblast behavior, inflammation, extracellular-matrix turnover, and cell migration. Recent work in hyperglycemic endothelial cells is particularly relevant: PBM improved proliferation, migration, and tubulogenesis through coordinated PDGF, VEGF, and TGF-β signaling, while other experimental studies implicate focal-adhesion pathways, extracellular-matrix remodeling, and inflammatory regulation [7,8,9]. These observations argue against a single-mediator model of PBM-induced repair.

An important gap nevertheless remains. Most mechanistic studies of PBM in diabetes have used cutaneous wound models, while comparatively little is known about the angiogenic response of oral mucosa. In addition, VEGF is commonly measured at a single time point and interpreted as evidence of enhanced angiogenesis. Such an approach overlooks the inherently dynamic nature of wound repair: a high VEGF concentration early after injury may have a very different biological meaning from the same concentration during later tissue organization. Examining VEGF alongside semiquantitative histopathological scores of vascularization, fibroblasts, granulation tissue, and inflammatory injury, together with binary mucosal continuity, may help characterize the relationship between the biochemical response and tissue-level repair across healing phases [1,2,3,10].

A previous report from this experimental cohort examined macroscopic ulcer closure and systemic oxidative-stress markers and showed treatment-associated differences in both outcomes [5]. Tissue VEGF and histopathological outcomes were not reported or analyzed in that publication. The present study therefore re-examines the ulcer-bearing cohort from a distinct tissue-level perspective, asking whether the temporal VEGF response remains aligned with histopathological repair across successive healing phases.

We therefore investigated whether diabetes and photobiomodulation alter the temporal pattern of tissue VEGF during oral mucosal repair and whether this biochemical response remains aligned with the developing histological phenotype. Particular attention was given to two questions: whether the effects of diabetes and PBM on VEGF change across healing phases, and whether VEGF remains associated with tissue repair after accounting for healing day, metabolic status, and treatment. We expected diabetes to blunt and PBM to enhance the early VEGF response. We further hypothesized that, as healing progressed, tissue organization would become increasingly dissociated from contemporaneous VEGF concentrations, reflecting the contribution of multiple reparative processes beyond VEGF alone.

## 2. Results

### 2.1. Analytical Cohort and Outcome Structure

The analysis included all 72 ulcer-bearing animals with available tissue VEGF and histopathological data, distributed across four experimental conditions and three healing time points (n = 6 per metabolic-status × treatment × time cell). Tissue VEGF served as the principal biochemical outcome, while histopathological analyses included individual features and exploratory inflammation/injury and tissue repair scores.

### 2.2. Tissue VEGF Exhibited Phase-Dependent Responses to Diabetes and LLLT

**Figure 1:**
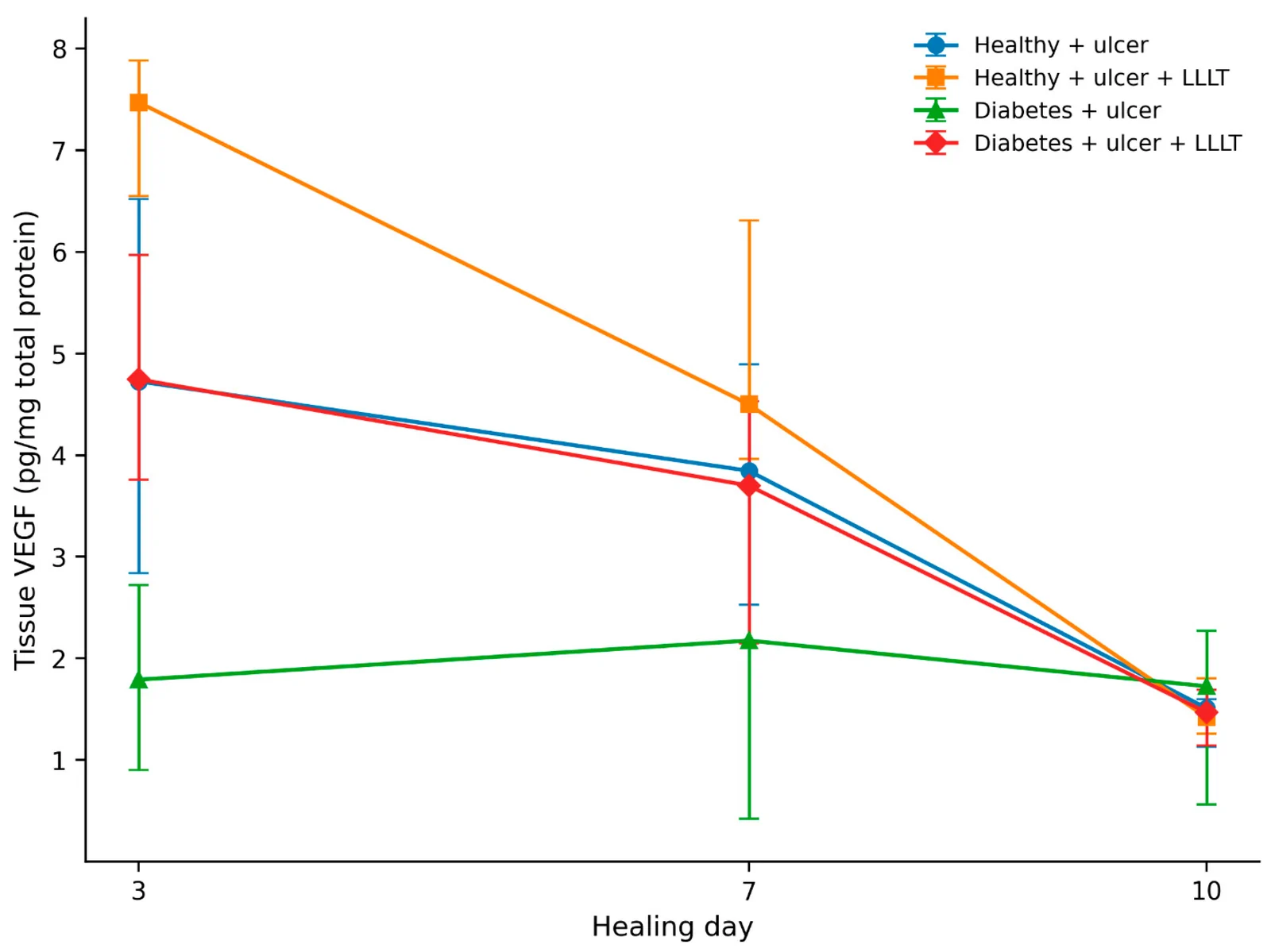
**Temporal dynamics of tissue VEGF across experimental groups.** Points represent median tissue VEGF concentrations, and vertical bars represent the observed minimum–maximum range at each healing day. VEGF concentrations are expressed as pg/mg total protein. Abbreviations: LLLT, low-level laser therapy; VEGF, vascular endothelial growth factor.

Tissue VEGF differed markedly across experimental conditions during the early healing phase (Figure 1). On day 3, median VEGF values were 4.72 pg/mg total protein in healthy ulcerated animals, 7.47 in healthy LLLT-treated animals, 1.79 in diabetic ulcerated animals, and 4.75 in diabetic LLLT-treated animals. Group separation was smaller on day 7 and no longer evident by day 10.

In the factorial log-VEGF model, significant diabetes × day (*p* = 0.0017) and LLLT × day (*p* = 0.0010) interactions demonstrated that the effects of metabolic status and treatment varied across healing phases. The diabetes × LLLT interaction (*p* = 0.115) and the three-way diabetes × LLLT × day interaction (*p* = 0.300) were not significant (Table 1).

### 2.3. Day-Specific VEGF Contrasts Confirmed Early LLLT and Diabetes Effects

Day-specific model contrasts showed the largest LLLT effect on day 3, when treated animals had 2.13-fold higher VEGF than untreated animals after adjustment for diabetes status (95% CI 1.63–2.78; Holm-adjusted *p* < 0.001). The estimated treatment ratio decreased to 1.65 on day 7 (95% CI 1.06–2.57; adjusted *p* = 0.052), and was 1.01 on day 10 (95% CI 0.77–1.33; adjusted *p* = 0.929).

**Figure 2:**
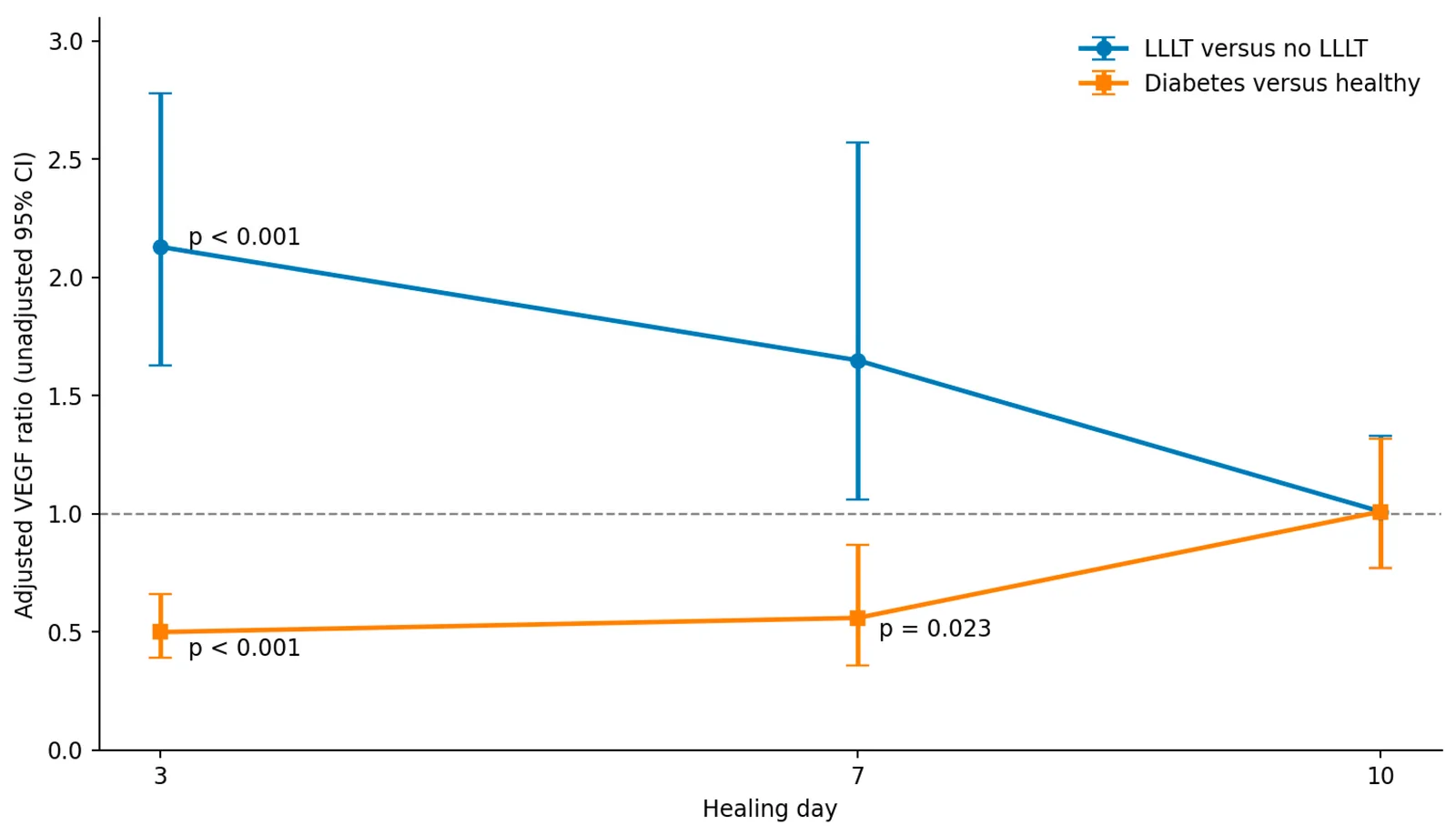
**Day-specific effects of LLLT treatment and diabetes status on tissue VEGF.** Back-transformed model-derived contrasts from the factorial log-VEGF model. Error bars denote unadjusted 95% confidence intervals. Holm-adjusted *p* values are indicated only for significant comparisons: LLLT versus no LLLT on day 3 (*p* < 0.001), diabetes versus healthy on day 3 (*p* < 0.001), and diabetes versus healthy on day 7 (*p* = 0.023). The day-7 LLLT contrast is not significant after Holm correction (*p* = 0.052). The dashed horizontal line denotes a ratio of 1. Abbreviations: CI, confidence interval; LLLT, low-level laser therapy; VEGF, vascular endothelial growth factor.

Diabetes showed the opposite day-specific pattern. Compared with healthy animals, diabetic animals had approximately half the VEGF concentration on day 3 (ratio 0.50, 95% CI 0.39–0.66; adjusted *p* < 0.001) and lower VEGF on day 7 (ratio 0.56, 95% CI 0.36–0.87; adjusted *p* = 0.023), whereas no difference was observed on day 10 (ratio 1.01, 95% CI 0.77–1.32; adjusted *p* = 0.960) (Table 2; Figure 2).

Supportive day-stratified Mann–Whitney analyses yielded the same overall temporal pattern: diabetes-associated differences remained significant on days 3 and 7, while the LLLT-associated difference remained significant only on day 3 after Holm correction (Supplementary Table S1).

### 2.4. Histopathological Differences Varied Across Healing Days

**Figure 3:**
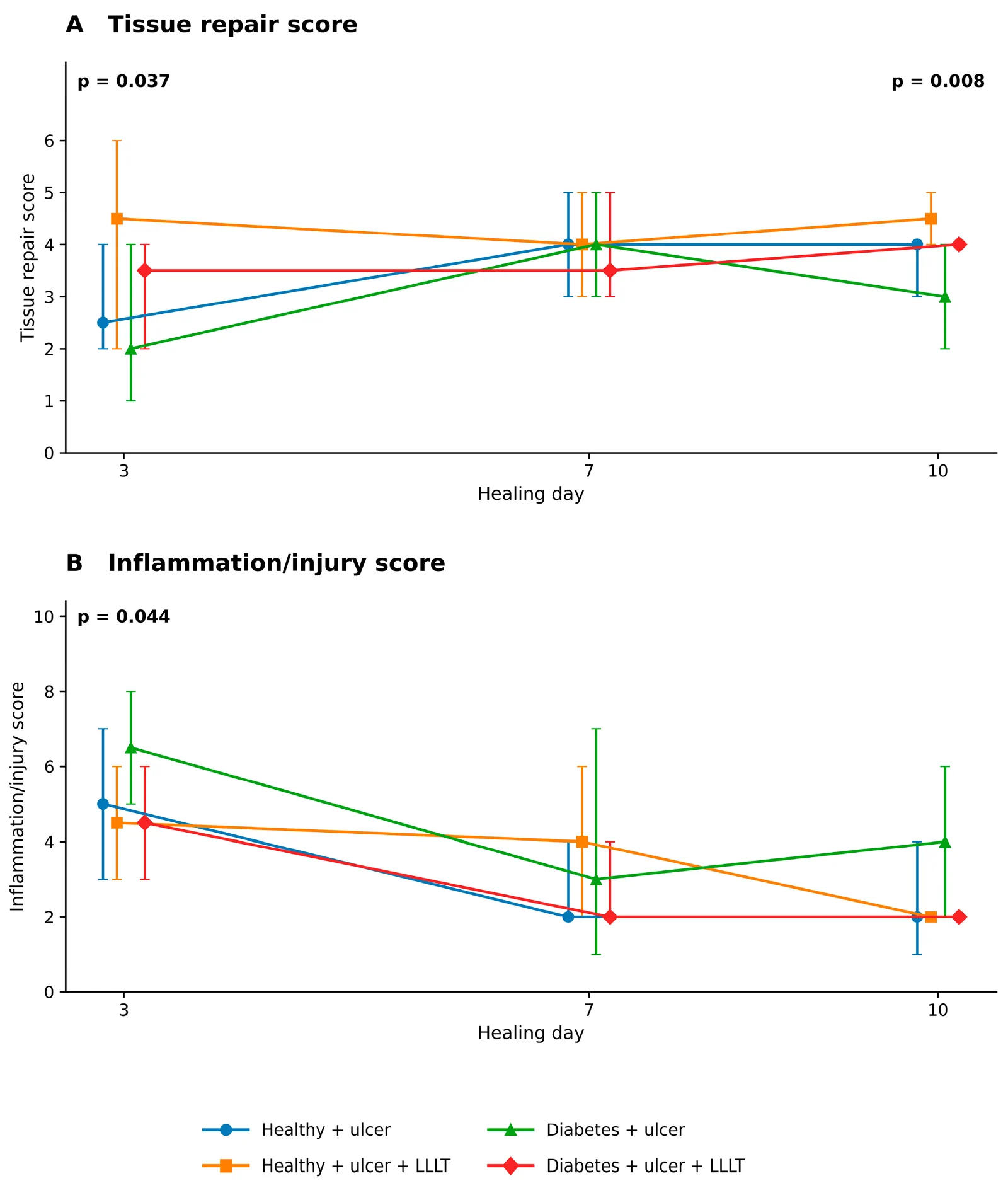
**Exploratory histopathological composite scores by metabolic status, LLLT treatment, and healing day.** (**A**) Tissue repair score (range 0–9), comprising vascularization, fibroblasts, and granulation tissue. (**B**) Inflammation/injury score (range 0–15), comprising necrosis, neutrophils, macrophages, lymphocytes, and plasma cells. Symbols represent group medians, and error bars indicate the observed minimum–maximum range (n = 6 animals per group and healing day). Lines connect group medians from independent animal cohorts assessed at successive healing days. Indicated *p* values represent exploratory overall Kruskal–Wallis comparisons among the four experimental groups within each healing day. These analyses were not adjusted for multiplicity; significant omnibus results do not establish significant pairwise differences. Abbreviation: LLLT, low-level laser therapy.

Histopathological group differences were most evident on day 3. Significant overall differences were observed for vascularization (Kruskal–Wallis *p* = 0.042), the inflammation/injury score (*p* = 0.044), and the exploratory tissue repair score (*p* = 0.037). The temporal profiles of the two composite scores are shown in Figure 3; complete descriptive and Kruskal–Wallis results are provided in Supplementary Tables S2 and S3A. These histopathological comparisons were exploratory and were not multiplicity-adjusted; nominal *p* values are therefore interpreted descriptively.

The exploratory tissue repair score also differed among groups on day 10 (*p* = 0.008), despite the absence of corresponding group differences in tissue VEGF at that time point. Mucosal continuity was analyzed separately as a binary endpoint. There was no outcome variability on day 3, and no significant between-group differences were detected on day 7 (Fisher–Freeman–Halton exact *p* = 0.524) or day 10 (exact *p* = 0.878) (Supplementary Table S3B).

**Figure 4:**
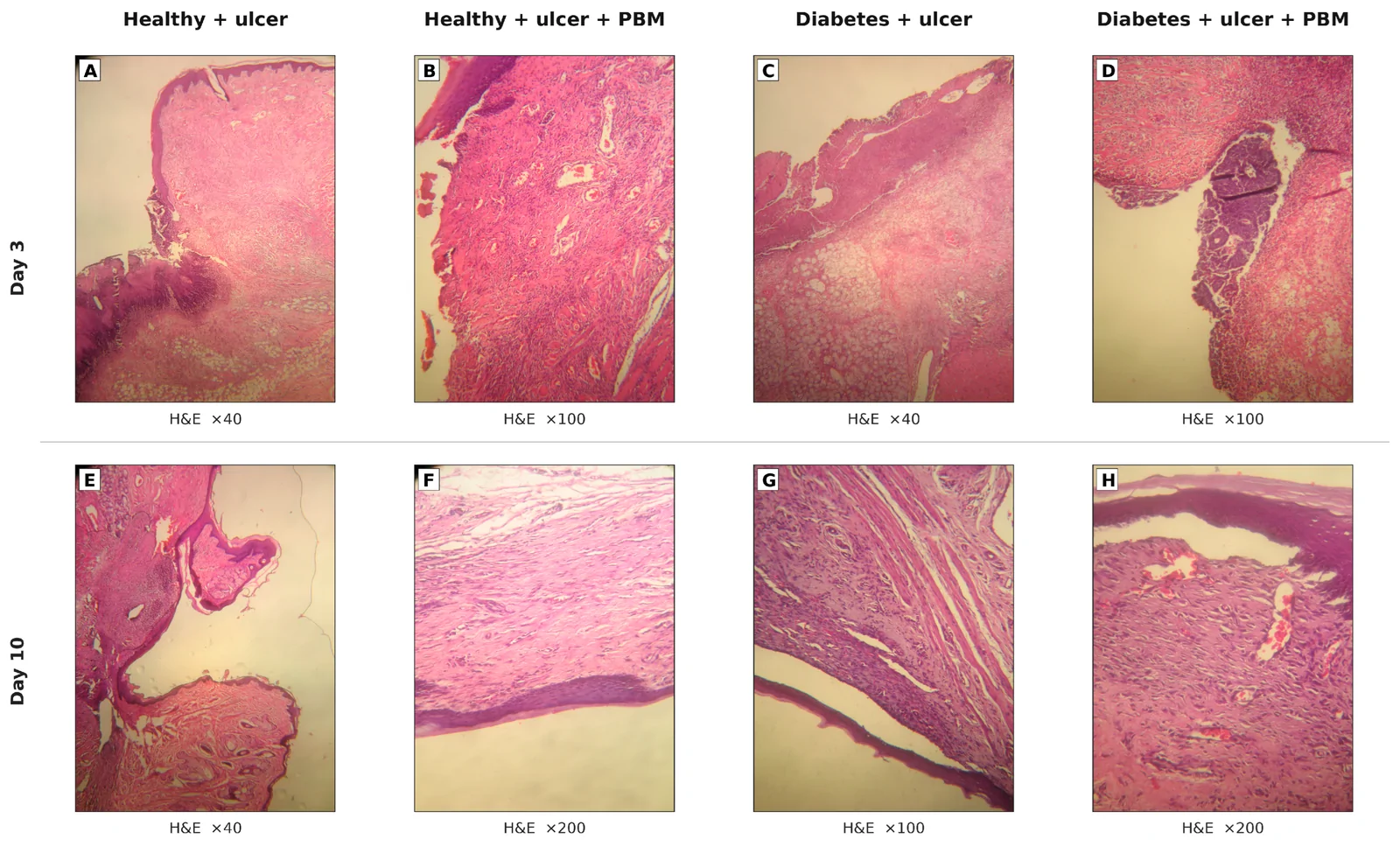
**Representative histopathological features of oral mucosal wound healing in healthy and diabetic rats following LLLT.** Representative hematoxylin and eosin (H&E)-stained sections obtained on days 3 (**A**–**D**) and 10 (**E**–**H**) after oral mucosal ulcer induction. Panels (**A**,**E**) represent healthy ulcerated animals without treatment; (**B**,**F**), healthy ulcerated animals receiving LLLT; (**C**,**G**), diabetic ulcerated animals without treatment; and (**D**,**H**), diabetic ulcerated animals receiving LLLT. Original magnifications are indicated beneath each panel. Microphotographs were acquired using a Canon PowerShot S70 digital camera (Canon Inc., Tokyo, Japan) coupled to a Leica microscope. Calibrated scale bars could not be included because the original histological slides and microscope–camera calibration records were unavailable. Images are presented as representative qualitative illustrations and were not used for direct quantitative morphometric comparisons. Abbreviations: H&E, hematoxylin and eosin; LLLT, low-level laser therapy.

Representative H&E micrographs illustrating the early injury/inflammatory phenotype on day 3 and the later reparative phenotype on day 10 across the four experimental groups are shown in Figure 4. Complementary Masson trichrome sections illustrating tissue organization across the corresponding experimental groups and healing phases are provided in Supplementary Figure S2.

### 2.5. LLLT Remained Associated with Tissue Repair After Adjustment

In rank-based multivariable models adjusted for healing day, diabetes status, LLLT treatment, and tissue VEGF, LLLT was associated with a higher exploratory tissue repair score (β = 17.489, 95% CI 8.541 to 26.436; HC3 *p* < 0.001), while diabetes was associated with a lower score (β = −11.796, 95% CI −20.217 to −3.375; HC3 *p* = 0.006). VEGF itself was not independently associated with the composite repair score (β = −0.237, 95% CI −0.591 to 0.116; HC3 *p* = 0.189) (Table 3). Because outcomes were rank-transformed, these coefficients represent shifts in outcome rank rather than changes in the original histological score units.

Healing day was the principal correlate of inflammation/injury. Relative to day 3, inflammation/injury scores were lower on days 7 and 10 (both HC3 *p* < 0.001). VEGF was not independently associated with inflammation/injury (β = −0.004, 95% CI −0.318 to 0.310; HC3 *p* = 0.979).

In exploratory component-level models, LLLT was associated with greater vascularization (β = 14.095, 95% CI 5.671 to 22.519; HC3 *p* = 0.001) and higher fibroblast scores (β = 8.462, 95% CI 3.447 to 13.477; HC3 *p* < 0.001), while diabetes was associated with lower fibroblast scores (β = −7.061, 95% CI −12.174 to −1.948; HC3 *p* = 0.007). These component-level analyses were not adjusted for multiplicity and are therefore reported as supportive findings (Supplementary Table S4).

In post hoc sensitivity analyses, the adjusted association of LLLT with the exploratory tissue repair score remained positive after sequential exclusion of vascularization (β = 10.19, HC3 *p* = 0.0066), fibroblasts (β = 7.87, *p* = 0.0380), and granulation tissue (β = 15.38, *p* < 0.001). These analyses evaluate dependence on composite construction and do not constitute independent morphological validation (Supplementary Table S7 and Supplementary Figure S3).

### 2.6. Day-3 VEGF–Histology Associations Before and After Adjustment

On day 3, VEGF correlated positively with vascularization (Spearman ρ = 0.508, *p* = 0.011), fibroblast score (ρ = 0.495, *p* = 0.014), and tissue repair score (ρ = 0.486, *p* = 0.016), and negatively with inflammation/injury (ρ = −0.521, *p* = 0.009) (Supplementary Table S5).

After adjustment for diabetes status and LLLT treatment, none of these associations remained statistically significant: tissue repair *p* = 0.671, vascularization *p* = 0.734, fibroblast score *p* = 0.574, and inflammation/injury *p* = 0.830. Corresponding unadjusted and adjusted VEGF–histology associations are summarized in Supplementary Table S5 and Supplementary Figure S1.

## 3. Discussion

The main observation of this study was a clear temporal separation between the early VEGF response and the later tissue-repair phenotype. Diabetes was associated with lower tissue VEGF during the early healing phase, whereas photobiomodulation produced the opposite pattern; both differences diminished as healing progressed and were no longer detectable by day 10. In contrast, the association between photobiomodulation and exploratory tissue repair persisted after adjustment for healing phase, metabolic status, and contemporaneous tissue VEGF. VEGF itself did not retain an independent association with the composite repair phenotype. Taken together, these findings indicate that LLLT is associated with an early increase in tissue VEGF, while subsequent differences in exploratory histopathological repair scores are not independently explained by contemporaneous VEGF concentrations in the adjusted models.

The interaction with healing day is biologically important because VEGF is not expected to remain constant throughout wound repair. Its role is greatest during the transition into proliferative and angiogenic activity, when endothelial recruitment and microvascular expansion support granulation tissue formation. As tissue organization advances, processes such as extracellular-matrix remodeling, vascular maturation, fibroblast responses, and epithelial restoration may become increasingly important. However, these processes were not directly characterized at the cellular or molecular level in the present study. The pronounced group separation on day 3, followed by convergence by day 10, is therefore consistent with VEGF functioning as a phase-sensitive signal rather than as a stable marker of overall healing quality [1,10].

The diabetic phenotype was characterized primarily by attenuation of the early VEGF response rather than by persistent suppression throughout the 10-day observation period. The approximately 50% lower adjusted VEGF concentration on day 3, with a residual difference on day 7 but not day 10, indicates that diabetes was associated with changes in both the magnitude and temporal pattern of tissue VEGF concentrations. This pattern is compatible with the endothelial dysfunction, disturbed hypoxia sensing, persistent inflammatory activity, and impaired stromal-cell responses described in diabetic wounds [4,10,11,12]. Importantly, the convergence of VEGF concentrations by day 10 argues against interpreting diabetes simply as a uniform reduction in VEGF production across all healing phases.

Photobiomodulation showed the mirror image of this temporal pattern. Its effect on VEGF was strongest on day 3, weaker on day 7, and absent by day 10. Importantly, neither the diabetes × PBM interaction nor the three-way interaction with healing day was significant. The study therefore did not provide statistical evidence that the VEGF response to PBM differed between diabetic and healthy animals. This distinction is relevant because current evidence increasingly places PBM within a network of endothelial, stromal, inflammatory, mitochondrial, and matrix-related responses rather than within a single growth-factor pathway [7,8,9,13].

The histological data reinforce the idea that the VEGF response and tissue repair are temporally related but not interchangeable. PBM remained associated with a higher exploratory tissue repair score after adjustment for healing day, diabetes, and VEGF, and the component-level analyses pointed in the same direction for vascularization and fibroblast scores. The directionally consistent findings for the composite and selected component scores help characterize which histopathological features contributed to the overall association, although these component-level results do not constitute independent validation of the composite measure. At the same time, VEGF was not independently associated with the composite repair score. The contrast was most apparent on day 10: tissue-repair differences were still detectable after tissue VEGF concentrations had converged across groups. The early VEGF pattern is consistent with a transient change in the local biochemical environment, whereas the later histopathological findings suggest treatment-associated differences in tissue repair that are not fully captured by contemporaneous VEGF measurements. The cellular and molecular mechanisms underlying these differences remain to be established [7,8,14].

The day-3 analyses provide an additional caution against interpreting VEGF as the sole driver of the histological response. Before adjustment, higher VEGF accompanied greater vascularization, more fibroblasts, higher tissue repair, and less inflammatory injury. These associations disappeared after accounting for diabetes and PBM treatment. This pattern indicates that VEGF and histological repair were strongly embedded within the same experimental context. The present cross-sectional tissue analyses cannot establish formal mediation or a causal pathway from PBM through VEGF to histological repair; rather, they show that VEGF did not remain an independent explanatory factor once metabolic status and treatment were considered. Clinical findings also illustrate the importance of sampling compartment and timing: in a randomized trial involving diabetic foot ulcers, Torkaman et al. reported lower circulating VEGF after PBM than after placebo despite greater wound-area reduction; these serum findings should not be directly equated with local tissue VEGF measured in the present rat model [15]. More broadly, systematic evidence on VEGF-directed approaches in diabetic wounds also underscores that VEGF biology is relevant but does not by itself define the complete healing phenotype [16].

This interpretation fits current models of PBM biology. PBM can modify mitochondrial and redox signaling alongside endothelial, fibroblastic, inflammatory, and extracellular-matrix responses [7,8,9,13,14,17,18,19]. VEGF may therefore be considered one measured component of the early response rather than a sufficient explanation for the observed histopathological repair scores. The persistence of the association between LLLT treatment and the exploratory tissue repair score after VEGF adjustment is compatible with the involvement of additional processes, although these processes were not directly investigated in the present study.

In a broader biophysical context, Klabukov et al. have discussed how photonic and acoustic modalities of non-ionizing radiation may interact with extracellular-matrix properties and tissue responses, including proposed synergistic effects when different modalities are combined [20]. This framework provides conceptual context for the multifactorial nature of responses to physical stimulation, but it should not be interpreted as evidence of multimodal synergy in the present experiment, which investigated PBM alone.

The oral location also matters. Oral mucosal wounds differ biologically from cutaneous wounds in epithelial turnover, inflammatory kinetics, microbial exposure, salivary signaling, extracellular-matrix composition, vascular responses, and scar formation [1,2,3]. These differences may alter both the timing and magnitude of angiogenic responses. Diabetes adds endothelial, inflammatory, and redox disturbances to this otherwise rapidly repairing environment [10,11]. The present findings therefore extend PBM research beyond the more frequently studied cutaneous diabetic wound and suggest that temporal angiogenic–tissue repair coordination is also relevant to oral mucosal repair.

Several design features strengthen the interpretation of these findings. The balanced 2 × 2 × 3 structure allowed diabetes, PBM, and healing phase to be examined within a single factorial framework. VEGF was measured directly in ulcer tissue and normalized to total protein, while histological assessment captured vascular, fibroblastic, inflammatory, necrotic, granulation, and epithelial features. Importantly, the analysis did not rely solely on unadjusted pairwise comparisons: factorial models, robust inference, and adjusted day-3 analyses were used to test whether the apparent VEGF–histology relationships persisted after accounting for experimental group. Unlike the parent study, which addressed macroscopic closure and systemic redox status, the present analysis tests a distinct tissue-level question: whether the temporal VEGF response remains coupled to tissue repair across successive healing phases. All VEGF, histopathological, composite-score, and integrated VEGF–histology outcomes reported here were previously unpublished [5].

Several limitations should be considered. First, this was a secondary analysis of an existing experiment, and the available sample size was fixed by the parent design. No formal a priori power calculation had been performed for the parent experiment; the original group size was based on comparable oral wound-healing studies, feasibility considerations, and established experimental precedent [5]. Although the balanced factorial structure was informative for the observed treatment-, metabolic-status-, and time-dependent patterns, n = 6 per cell limits precision for small higher-order interactions; non-significant interaction terms should therefore not be interpreted as evidence of equivalence. Second, most histological variables were semiquantitative scores, while mucosal continuity was recorded as a binary indicator; the derived inflammation/injury and tissue repair composites were exploratory rather than validated measurement scales. Third, VEGF was measured in whole-tissue homogenates and cannot identify cellular source, quantify neovascularization directly, or establish receptor-level or downstream pathway activation. Additional endothelial and angiogenic markers such as CD31, VEGFR2 activation, HIF-1α, or complementary growth factors would be required for a pathway-level mechanistic analysis. In addition, the archived VEGF assay records did not document replicate measurements or assay-specific intra- and inter-assay coefficients of variation, limiting retrospective assessment of analytical precision. Fourth, the archived records did not specify the exact number of microscopic fields evaluated per animal. Fifth, although the parent study and its accompanying ARRIVE documentation reported random allocation using computer-generated random numbers, the original randomization sequence itself, details of allocation concealment, and measures used to control cage-position or treatment-order effects were not available in the archived dataset used for the present secondary analysis. Sixth, VEGF and histology were assessed in terminal samples from different animals at each healing day; the design therefore cannot demonstrate that an early VEGF level in an individual animal predicts its later histological outcome or establish temporal mediation. Only male rats were included in the parent experiment; consequently, potential sex-related differences in angiogenic, histological, or photobiomodulation responses could not be assessed. Finally, the findings arise from an alloxan-induced rat model and require validation before translation to human diabetic oral wounds. Incomplete records of anesthesia dosing, euthanasia procedures, and assay-specific ELISA precision may have introduced unquantified procedural or analytical variability. Conventional H&E and Masson trichrome staining, together with blinded semiquantitative scoring, provide descriptive tissue-level information but do not establish specific immune-cell phenotypes, directly quantify neovascularization, or identify cellular sources of VEGF. Immunohistochemistry and calibrated digital morphometry could provide complementary evidence in future investigations. Neither was performed in the parent experiment, and the original slides are no longer available for additional staining or rescanning.

## 4. Materials and Methods

### 4.1. Study Design and Relationship to the Previously Published Cohort

This study is a secondary analysis of a previously reported in vivo experiment evaluating low-level laser therapy in healthy and diabetic rats with experimentally induced oral mucosal ulcers [5]. The parent experiment included 108 male Wistar rats distributed across six experimental conditions. The present analysis was restricted to the 72 ulcer-bearing animals because the current research question concerned temporal biochemical and histopathological responses within ulcer-bearing tissue. The parent publication did not report or analyze the archived histopathological assessments or tissue VEGF measurements. These materials and measurements were available from the original experiment but were outside the scope of the previous publication, which focused on macroscopic wound closure and systemic oxidative-stress markers. The present secondary analysis therefore addresses previously unpublished tissue VEGF measurements, histopathological variables, exploratory composite histological scores, and their temporal relationships.

The analytical design comprised four conditions—healthy + ulcer, healthy + ulcer + LLLT, diabetes + ulcer, and diabetes + ulcer + LLLT—evaluated on days 3, 7, and 10 after ulcer induction (n = 6 per metabolic-status × treatment × time cell). No animals or data points from the eligible ulcer-bearing cohort were excluded, and no additional animals were used.

### 4.2. Experimental Animals, Housing, Allocation, and Ethics

Adult male Wistar rats (260–330 g), originating from litters obtained from the Military Medical Academy, University of Defence, Belgrade, were housed in the Vivarium of the Faculty of Medicine, University of Niš under controlled temperature (21–23 °C), a 12 h light/dark cycle, and free access to food and water. Animals were housed six per cage. The present analysis included the 72 ulcer-bearing animals from the parent experiment, with six animals in each metabolic-status × treatment × time combination. The individual animal was considered the experimental unit. No formal a priori power calculation was performed for the parent experiment. As previously reported [5], the parent sample size of 18 animals per experimental group was based on comparable experimental oral wound-healing studies, feasibility considerations, and established use of days 3, 7, and 10 to represent successive phases of wound repair. Six animals from each group were evaluated at each time point. The present secondary analysis did not involve a new sample-size determination; its analytical sample was fixed by the 72 ulcer-bearing animals available from the parent experimental design.

The protocol was approved by the Ethics Committee of the Faculty of Medicine in Priština (No. 05-1594, 24 September 2014) and the Ethics Committee of the Faculty of Medicine, University of Niš (No. 01-2625-1; 8 April 2014). Procedures complied with applicable Serbian animal-welfare legislation and the European Convention for the Protection of Vertebrate Animals Used for Experimental and Other Scientific Purposes (ETS 123). Reporting of the present analysis followed ARRIVE 2.0 principles [21].

Animal identification was performed using a yellow marking pen applied to the fur at different anatomical locations to facilitate visual differentiation of individual animals. The manufacturer and formulation of the marking material were not recoverable from the archived documentation.

Animals were reported in the parent study and its accompanying ARRIVE documentation as randomly allocated to experimental groups using computer-generated random numbers after confirmation of diabetic status, with allocation conducted by a researcher not involved in outcome assessment. The original randomization sequence itself and details of allocation concealment are not available in the archived dataset used for the present secondary analysis. As reported for the parent experiment, personnel administering LLLT were aware of treatment allocation but were not involved in outcome assessment. For the present analysis, histopathological assessment and VEGF quantification were performed blinded to experimental group. Animals were monitored daily for general condition, wound status, and signs of distress. No humane endpoints were reached and no adverse events were recorded.

### 4.3. Induction and Verification of Diabetes Mellitus

Diabetes was induced according to the parent experimental protocol [5]. After a 12 h fast, animals assigned to diabetic groups received a single intraperitoneal injection of alloxan monohydrate (150 mg/kg; Abcam Inc. Waltham, MA, USA) dissolved in physiological saline. A 10% glucose solution was provided for the first 24 h after injection to reduce the risk of early hypoglycemia. Tail-vein glucose was measured 72 h later using an Accu-Chek Performa glucometer (Roche Diagnostics, Mannheim, Germany), and animals with glucose >11.1 mmol/L (200 mg/dL) were considered diabetic. The oral-wound phase began four weeks after diabetes induction.

### 4.4. Oral Mucosal Ulcer Induction

Four weeks after diabetes induction, standardized oral mucosal ulcers were created under general anesthesia, following the parent experimental protocol [5] and informed by established rat intraoral wound models [22]. After disinfection with 0.12% chlorhexidine, a 6 mm circular defect was created on the right buccal mucosa with a No. 15 scalpel blade, extending to the muscle layer. Hemostasis was achieved by gentle compression with sterile gauze. The archived records identified Ketamidor (Richter Pharma AG, Wels, Austria) as the anesthetic agent but did not contain a recoverable dose; no retrospective dose estimate was therefore introduced.

### 4.5. Photobiomodulation Protocol

Photobiomodulation was administered according to the previously reported protocol for this experimental cohort [5], with reference to earlier experimental work on LLLT in rat oral mucosal repair [23]. A Scorpion Dental Optima device (Optica Laser, Sofia, Bulgaria) operating at 630–660 nm delivered continuous-wave irradiation at 7 mW through a 0.196 cm^2^ probe. The fluence was 6 J/cm^2^, corresponding to approximately 1.18 J per treatment session, 168 s exposure, and an irradiance of approximately 35.7 mW/cm^2^. The probe was held 1 mm from the wound surface using a fixed holder. Treatment began immediately after ulcer creation and was repeated every other day during the 10-day observation period. The available wavelength, operating mode, power, irradiance, fluence, exposure time, beam area, application distance, and treatment schedule are reported explicitly to support reproducibility, consistent with current recommendations for laser-therapy reporting [24]. The laser probe had a flat-top beam profile, providing a nominally uniform spatial irradiance distribution across the treatment area. The reported irradiance represents the nominal average value calculated from the output power and nominal probe area. The spatial irradiance distribution was not independently measured during the original experiment.

### 4.6. Sacrifice and Tissue Sampling

Adult animals were euthanized on days 3, 7, or 10 after ulcer induction (n = 6 per experimental condition at each time point). Body weight and glycemia were recorded on the day of sacrifice. The archived documentation describes euthanasia by anesthetic overdose but does not contain sufficient information to identify reliably the agent, dose, and route used specifically for this procedure. These details were therefore not reconstructed retrospectively.

Immediately after euthanasia, the ulcer-bearing tissue was divided for histopathological and biochemical analyses. Histology samples were fixed in 10% buffered formalin for 48 h, whereas tissue intended for VEGF analysis was placed in pre-weighed tubes and stored at −80 °C until processing.

### 4.7. Tissue Processing and Histopathological Assessment

Formalin-fixed specimens were processed routinely for paraffin histology. After graded ethanol dehydration and benzene clearing, tissues were paraffin-embedded at 58 °C using a Leica EG1150H system (Leica Biosystems, Wetzlar, Germany) and sectioned at 4–5 µm with a Leica RM2125RT rotary microtome (Leica Biosystems, Wetzlar, Germany).

Sections were stained with hematoxylin and eosin and Masson trichrome. Histological examination and image acquisition were performed using a Leica microscope coupled to a Canon PowerShot S70 digital camera. The original glass slides and spatial calibration records were no longer available, precluding reliable reconstruction of micrometer scale bars.

The archived histopathological assessment included necrosis, neutrophils, macrophages, lymphocytes, plasma cells, vascularization, fibroblasts, and granulation tissue. Each feature was assessed semiquantitatively according to the proportion of the examined histological field showing the respective finding, using a four-point scale: 0 = absent (0%), 1 = mild (≤30%), 2 = moderate (31–70%), and 3 = severe (≥71%). The selection of these histopathological features was consistent with previously published approaches to the assessment of experimental intraoral mucosal wounds that included the same or closely related parameters [22]. A related percentage-based grading approach was described in wound-healing research [25], but that report used the thresholds for macroscopic wound features and does not directly validate their use across the microscopic parameters measured here. The archived four-point scale is therefore presented as a study-specific semiquantitative instrument, not as a formally validated histological scale. Mucosal continuity was recorded separately as a binary histopathological variable (0 = absent; 1 = present), reflecting restoration of continuous mucosal coverage across the wound.

Published experimental wound-healing studies provide methodological precedents for ordinal microscopic grading. Abramov et al. used a modified histological wound-healing assessment covering inflammatory and reparative processes and reported interobserver agreement [26]. Nikolaou et al. applied percentage-defined categories to leukocyte infiltration in H&E-stained rat burn wounds [27]. However, their thresholds and outcome definitions differ from the present study. Neither publication validates the exact 0%, ≤30%, 31–70%, and ≥71% thresholds for all eight outcomes examined here; the present thresholds are therefore treated as study-specific and the derived scores as exploratory.

Two pathologists independently scored all histological specimens. Both were blinded to all accompanying study information, including metabolic status, LLLT treatment, healing-day assignment, VEGF measurements, and other study outcomes, and neither had access to the other pathologist’s ratings. For each semiquantitative histological feature, the mean of the two independent ratings was used in the statistical analyses. The archived records did not specify the exact number of microscopic fields or sections evaluated per animal.

Representative H&E micrographs from the archived histopathological documentation were selected to illustrate the tissue phenotypes characterized by the semiquantitative histopathological assessment; all statistical analyses were based on the original histopathological scores rather than on the representative images. Masson trichrome sections are provided in Supplementary Figure S2 to illustrate tissue organization across experimental groups and healing phases. The representative images are qualitative illustrations; they were not used for new quantitative morphometry.

### 4.8. Tissue VEGF Quantification

Ulcer tissue allocated to biochemical analysis was weighed, homogenized in TRIS buffer to produce a 10% homogenate, and centrifuged at 14,000 rpm for 10 min at 4 °C. Supernatants were collected individually and stored at −80 °C until analysis.

Tissue VEGF was quantified by an investigator blinded to experimental allocation using the Rat VEGF Quantikine ELISA Kit (R&D Systems, Minneapolis, MN, USA), following the manufacturer’s protocol. Absorbance was measured at 450 nm with a Multiskan Ascent microplate reader, and VEGF concentrations were derived from the assay standard curve.

Total protein concentration was determined by the Lowry method [28] using a spectrophotometric calibration curve. Tissue VEGF concentrations were normalized to total protein and expressed as pg/mg total protein. For the present analysis, tissue VEGF was interpreted as a biochemical marker of the local angiogenic environment rather than as a direct measure of neovascularization or evidence of VEGF-receptor pathway activation. Archived records did not document whether samples were assayed in duplicate or provide intra- or inter-assay coefficients of variation; these parameters were therefore not reconstructed.

### 4.9. Derived Histopathological Scores

For the present secondary analysis, biologically related semiquantitative histopathological variables were combined into two exploratory composite scores. The inflammation/injury score comprised necrosis, neutrophils, macrophages, lymphocytes, and plasma cells (range 0–15). The tissue repair score comprised vascularization, fibroblasts, and granulation tissue (range 0–9). All composite components were equally weighted because the source variables used the same four-point semiquantitative scale and no validated weighting scheme existed for this model. These composites were used as biologically informed summary measures rather than as validated histopathological instruments, and their results were interpreted alongside the corresponding individual components. Mucosal continuity was not incorporated into either composite score because it was recorded as a separate binary histopathological endpoint (0 = absent; 1 = present). For each animal, inflammation/injury = necrosis + neutrophils + macrophages + lymphocytes + plasma cells (0–15), and tissue repair = vascularization + fibroblasts + granulation tissue (0–9). Each term was the mean of the two blinded pathologists’ ordinal ratings (0–3) for that component. Accordingly, the composites are exploratory rather than validated instruments. The score construction is summarized in Supplementary Table S6.

### 4.10. Statistical Analysis

The present statistical analysis was designed to reflect the 2 × 2 × 3 factorial structure of the experiment, the small number of animals per cell (n = 6), and the semiquantitative and binary structure of the histopathological data. Tissue VEGF was designated as the principal biochemical outcome, with the factorial VEGF model serving as the principal inferential analysis. Histopathological composites and component-level models were considered exploratory.

VEGF values were summarized using medians and observed ranges. Overall differences among the four ulcer-bearing groups within each healing day were assessed using the Kruskal–Wallis test. Day-stratified marginal comparisons evaluated diabetes status across treatment groups and LLLT treatment across metabolic-status groups using the Mann–Whitney U test. Within each contrast family, *p* values across the three healing days were adjusted using the Holm procedure. Additional fully stratified pairwise comparisons were considered exploratory and are reported in the Supplementary Material.

To formally assess time-dependent treatment and metabolic effects on VEGF, VEGF concentrations were log-transformed and analyzed in a factorial linear model containing DM status, LLLT treatment, healing day as a categorical factor, all two-way interactions, and the DM × LLLT × day interaction. Heteroskedasticity-consistent HC3 standard errors were used. Day-specific model contrasts were estimated on the log scale and back-transformed to VEGF ratios with 95% confidence intervals.

Histopathological variables and composite scores were summarized according to their measurement scale. Semiquantitative histopathological features and composite scores were reported as medians with minimum and maximum values. Overall group differences within each healing day were assessed using Kruskal–Wallis tests. Associations between VEGF and selected semiquantitative histological outcomes were explored using Spearman rank correlations, with particular emphasis on day 3. Mucosal continuity was treated separately as a binary endpoint and summarized as the number and proportion of animals with restored continuity within each experimental group and healing day. Because of the small number of animals per cell and sparse outcome distributions, between-group comparisons within individual healing days were performed using the Fisher–Freeman–Halton exact test. No multivariable model was fitted for this secondary binary endpoint.

Because the remaining histopathological outcomes were semiquantitative variables with sparse category distributions within individual experimental cells, adjusted analyses were performed on rank-transformed outcomes using linear models with HC3 robust covariance estimates. Principal models included diabetes status, LLLT treatment, healing day, and rank-transformed VEGF. Component-level analyses used the same framework and were interpreted as exploratory; no multiplicity correction was applied across these supportive models. To examine whether day-3 VEGF–histology associations were independent of experimental group, additional models restricted to day 3 (n = 24) were fitted as rank(outcome) ~ rank(VEGF) + diabetes status + LLLT treatment, also using HC3 covariance estimates. Coefficients from rank-based models quantify shifts in outcome rank rather than differences in the original histological scoring units and were therefore interpreted primarily according to direction, confidence intervals, and statistical evidence.

As post hoc exploratory sensitivity analyses, tissue repair scores were recalculated after excluding one component at a time (vascularization, fibroblasts, or granulation tissue). Each reduced score was rank-transformed and fitted using the same covariate structure (diabetes status, LLLT treatment, categorical healing day, and rank-transformed VEGF) and HC3 robust covariance estimator as the original adjusted model. Coefficients from differently constructed ranked outcomes should not be compared as original-score effect sizes. These exploratory analyses were not multiplicity-adjusted.

All tests were two-sided, with statistical significance defined as *p* < 0.05 and multiplicity-adjusted *p* values reported where prespecified multiple comparisons were performed. Given the exploratory nature of the derived histological scores, the limited subgroup size, and the semiquantitative structure of the underlying data, interpretation emphasized effect direction, temporal consistency, confidence intervals, and robustness after multivariable adjustment rather than isolated nominal *p* values.

The expanded secondary analyses were performed using IBM SPSS Statistics for Windows, version 30.0 (IBM Corp., Armonk, NY, USA). The factorial log-VEGF models and rank-based linear models used heteroskedasticity-consistent HC3 covariance estimates.

### 4.11. Transparency Regarding Cohort Reuse

The present study uses animals from the previously reported parent experiment [5], but all VEGF, histopathological, composite-score, and integrated VEGF–histology analyses reported here are previously unpublished. The prior publication addressed macroscopic wound closure and systemic oxidative-stress markers. No additional animals were used for the present analysis.

## 5. Conclusions

Diabetes and LLLT were associated with opposite, phase-dependent differences in tissue VEGF during oral mucosal wound healing. The LLLT-associated increase in VEGF was most pronounced early and was no longer detectable by day 10, whereas the exploratory tissue repair score remained associated with treatment after adjustment for healing day, diabetes status, and contemporaneous VEGF. These findings suggest that the treatment-associated histopathological repair phenotype is not fully explained by contemporaneous tissue VEGF concentrations. Further studies incorporating quantitative morphometry, immunohistochemical markers, and additional molecular analyses are needed to determine the cellular and mechanistic basis of this relationship.

## Figures and Tables

**Table 1:** Factorial model for log-transformed tissue VEGF.

Effect	*p* Value
Diabetes (DM)	<0.001
LLLT	<0.001
Day	<0.001
Diabetes × LLLT	0.115
Diabetes × Day	0.0017
LLLT × Day	0.0010
Diabetes × LLLT × Day	0.300

Model: log(VEGF) ~ diabetes × LLLT × day. Significant diabetes × day and LLLT × day interactions indicate that the effects of diabetes status and LLLT treatment on tissue VEGF varied across healing days. Abbreviations: DM, diabetes mellitus; LLLT, low-level laser therapy; VEGF, vascular endothelial growth factor.

**Table 2:** Model-derived day-specific contrasts for tissue VEGF.

Contrast	Day	Ratio	95% CI	Holm-Adjusted *p*
LLLT vs. no LLLT	3	2.13	1.63–2.78	<0.001
LLLT vs. no LLLT	7	1.65	1.06–2.57	0.052
LLLT vs. no LLLT	10	1.01	0.77–1.33	0.929
Diabetes vs. healthy	3	0.50	0.39–0.66	<0.001
Diabetes vs. healthy	7	0.56	0.36–0.87	0.023
Diabetes vs. healthy	10	1.01	0.77–1.32	0.960

Ratios were derived from day-specific contrasts of the factorial log-VEGF model and were back-transformed to the original ratio scale. Ratios >1 indicate higher VEGF in the first-listed group, whereas ratios <1 indicate lower VEGF in the first-listed group. The 95% confidence intervals are unadjusted; *p* values were adjusted across healing days 3, 7, and 10 within each prespecified contrast family using the Holm procedure. Abbreviations: CI, confidence interval; LLLT, low-level laser therapy; VEGF, vascular endothelial growth factor.

**Table 3:** Principal and supportive adjusted histopathological associations.

Outcome	Predictor/Contrast	β (Rank Scale), 95% CI	HC3 *p*
Tissue repair score	DM	−11.796 (−20.217 to −3.375)	0.006
Tissue repair score	LLLT	17.489 (8.541 to 26.436)	<0.001
Tissue repair score	Rank-transformed VEGF	−0.237 (−0.591 to 0.116)	0.189
Vascularization	LLLT	14.095 (5.671 to 22.519)	0.001
Fibroblasts	DM	−7.061 (−12.174 to −1.948)	0.007
Fibroblasts	LLLT	8.462 (3.447 to 13.477)	<0.001
Inflammation/injury score	Rank-transformed VEGF	−0.004 (−0.318 to 0.310)	0.979

Rank-based models included healing day, diabetes status, LLLT treatment, and rank-transformed VEGF. β coefficients are reported on the rank-transformed outcome scale with heteroskedasticity-consistent HC3 95% confidence intervals and *p* values. Positive coefficients indicate higher outcome ranks and should not be interpreted as changes in the original histological score units. Component-level histological analyses were exploratory and were not adjusted for multiplicity. The table summarizes the principal composite-score associations together with the component-level associations highlighted in the Results; complete component-level models are provided in Supplementary Table S4. Abbreviations: CI, confidence interval; DM, diabetes mellitus; LLLT, low-level laser therapy; VEGF, vascular endothelial growth factor.

## Data Availability

The data supporting the findings of this study are available from the corresponding author upon reasonable request.

## Supplementary Materials

The following supporting information can be downloaded at https://www.mdpi.com/article/10.3390/ijms27219395/s1.

## References

[1] Chuhuaicura P., Rodríguez-Niklitschek C., Oporto G.H., Salazar L.A. (2025). Distinct Molecular Mechanisms in Oral Mucosal Wound Healing: Translational Insights and Future Directions. Int. J. Mol. Sci..

[2] Waasdorp M., Krom B.P., Bikker F.J., van Zuijlen P.P.M., Niessen F.B., Gibbs S. (2021). The Bigger Picture: Why Oral Mucosa Heals Better Than Skin. Biomolecules.

[3] Overmiller A.M., Sawaya A.P., Hope E.D., Morasso M.I. (2022). Intrinsic Networks Regulating Tissue Repair: Comparative Studies of Oral and Skin Wound Healing. Cold Spring Harb. Perspect. Biol..

[4] Kamal R., Awasthi A., Pundir M., Thakur S. (2024). Healing the Diabetic Wound: Unlocking the Secrets of Genes and Pathways. Eur. J. Pharmacol..

[5] Đorđević N.S., Dragojević I.M., Ilić A.N., Stojanović N.M., Todić J.T., Puhalo Sladoje D., Stošović Kalezić I., Đorđević A.M., Jovanović R., Šubarić L. (2025). Effects of Low-Level Laser Therapy on Oral Mucosal Wound Healing and Systemic Oxidative Stress in Diabetic Rats: An In Vivo Experimental Study. Medicina.

[6] Radović K., Brković B., Roganović J., Ilić J., Milić Lemić A., Jovanović B. (2022). Salivary VEGF and Post-Extraction Wound Healing in Type 2 Diabetic Immediate Denture Wearers. Acta Odontol. Scand..

[7] Oliveira V.R.S., Varsani R., Zehra M., Dale C.S., Arany P. (2025). Endothelial Cell Responses to Photobiomodulation Treatments in Diabetic Wounds Are Mediated via Concerted PDGF, VEGF and TGF-β Signalling. Wound Repair Regen..

[8] Choi J., Ban M.J., Gil C.H., Hur S.S., Anggradita L.D., Kim M.K., Son J.W., Kim J.E., Hwang Y. (2025). Multispectral Pulsed Photobiomodulation Enhances Diabetic Wound Healing via Focal Adhesion-Mediated Cell Migration and Extracellular Matrix Remodeling. Int. J. Mol. Sci..

[9] Mgwenya T.N., Abrahamse H., Houreld N.N. (2025). Photobiomodulation Studies on Diabetic Wound Healing: An Insight into the Inflammatory Pathway in Diabetic Wound Healing. Wound Repair Regen..

[10] Huang K., Mi B., Xiong Y., Fu Z., Zhou W., Liu W., Liu G., Dai G. (2025). Angiogenesis during Diabetic Wound Repair: From Mechanism to Therapy Opportunity. Burn. Trauma.

[11] Ding S., Zhang X., Wang G., Shi J., Zhu J., Yan J., Wang J., Wu J. (2024). Promoting Diabetic Oral Mucosa Wound Healing with a Light-Responsive Hydrogel Adaptive to the Microenvironment. Heliyon.

[12] Jung J.P., Olutoye O.O., Prajapati T.J., Jung O.S., Yutzy L.D., Nguyen K.L., Wheat S.W., Huang J., Padon B.W., Faruk F. (2025). Sustained ROS Scavenging and Pericellular Oxygenation by Lignin Composites Rescue HIF-1α and VEGF Levels to Improve Diabetic Wound Neovascularization and Healing. Acta Biomater..

[13] Maghfour J., Mineroff J., Ozog D.M., Jagdeo J., Lim H.W., Kohli I., Anderson R., Kelly K.M., Mamalis A., Munavalli G. (2025). Evidence-Based Consensus on the Clinical Application of Photobiomodulation. J. Am. Acad. Dermatol..

[14] Oliveira V.R.S., Oliveira I.P., Alonso-Matielo H., Oliveira V.T., Kremer J.L., Casalverini M.C.D., Ribeiro F.Q., Maria-Engler S.S., Assis S.R., Teixeira M.J. (2025). Photobiomodulation Therapy in Diabetes: Benefits for Pain Relief, Quality of Life, and Wound Healing. Photochem. Photobiol..

[15] Torkaman G., Hoseini-Sanati M., Hedayati M., Mofid M., Iranparvar Alamdari M. (2024). Effects of Photobiomodulation Therapy on the Expression of Hypoxic Inducible Factor, Vascular Endothelial Growth Factor, and Its Specific Receptor: A Randomized Control Trial in Patients with Diabetic Foot Ulcer. Photobiomodul. Photomed. Laser Surg..

[16] Bang F.S., Leeberg V., Ding M., Dreyer C.H. (2024). The Effect of VEGF Stimulation in Diabetic Foot Ulcers: A Systematic Review. Wound Repair Regen..

[17] Lai F., Mai Y., Wang Z., Wang C., Xu S. (2026). Photobiomodulation Targets Mitochondrial Homeostasis for Diabetic Wound Healing. Tissue Eng. Part B Rev..

[18] Kurhaluk N., Tomin V., Kołodziejska R., Tkaczenko H. (2026). Photobiomodulation and Low-Level Laser Therapy as Complementary Strategies in Diabetes Treatment. Int. J. Mol. Sci..

[19] Pilar E.F.S., Brochado F.T., Schmidt T.R., Leite A.C., Deluca A.A., Mármora B.C., Siebert M., Wagner V.P., Martins M.D. (2024). Modulation of Gene Expression in Skin Wound Healing by Photobiomodulation Therapy: A Systematic Review in Vivo Studies. Photodermatol. Photoimmunol. Photomed..

[20] Klabukov I., Eygel D., Isaeva E., Kisel A., Isaev E.I., Potievskiy M., Atiakshin D., Shestakova V., Baranovskii D., Akhmedov B. (2025). Synergistic Effects of Non-Ionizing Radiation in the Targeted Modification of Living Tissues. Int. J. Mol. Sci..

[21] Percie du Sert N., Hurst V., Ahluwalia A., Alam S., Avey M.T., Baker M., Browne W.J., Clark A., Cuthill I.C., Dirnagl U. (2020). The ARRIVE Guidelines 2.0: Updated Guidelines for Reporting Animal Research. PLoS Biol..

[22] Al-Mobeeriek A. (2011). Effects of Myrrh on Intra-Oral Mucosal Wounds Compared with Tetracycline- and Chlorhexidine-Based Mouthwashes. Clin. Cosmet. Investig. Dent..

[23] Firat E.T., Dağ A., Günay A., Kaya B., Karadede M.I., Ersöz Kanay B., Ketani A., Evliyaoğlu O., Uysal E. (2014). The Effect of Low-Level Laser Therapy on the Healing of Hard Palate Mucosa and the Oxidative Stress Status of Rats. J. Oral Pathol. Med..

[24] Shurrab K. (2025). Proposed Guidelines for Reporting Parameters and Procedures of High- and Low-Level Laser Therapy in Medical Research Articles. Med. Devices.

[25] Conceição M., Gushiken L.F.S., Aldana-Mejía J.A., Tanimoto M.H., Ferreira M.V.d.S., Alves A.C.M., Miyashita M.N., Bastos J.K., Beserra F.P., Pellizzon C.H. (2022). Histological, Immunohistochemical and Antioxidant Analysis of Skin Wound Healing Influenced by the Topical Application of Brazilian Red Propolis. Antioxidants.

[26] Abramov Y., Golden B., Sullivan M., Botros S.M., Miller J.-J.R., Alshahrour A., Goldberg R.P., Sand P.K. (2007). Histologic characterization of vaginal vs. abdominal surgical wound healing in a rabbit model. Wound Repair Regen..

[27] Nikolaou C., Frountzas M., Schizas D., Pergialiotis V., Kapetanakis E.I., Kontzoglou K., Perrea D.N., Koniaris E., Kykalos S., Iliopoulos D. (2024). Could Fibrin Sealants (TISSEEL™) Be Effective in the Management of Burn Injuries? A Histopathological Study in Rats. Med. Sci..

[28] Lowry O.H., Rosebrough N.J., Farr A.L., Randall R.J. (1951). Protein Measurement with the Folin Phenol Reagent. J. Biol. Chem..

