# Supplementary material for: Photobiomodulation Modulates Temporal VEGF Dynamics and Tissue Repair During Diabetic Oral Mucosal Wound Healing in Rats
Source: Int J Mol Sci. 2026 Oct 22;27(21):9395. doi: 10.3390/ijms27219395 (PMC13762798; doi:10.3390/ijms27219395)
Supplement: Supplementary file 1 [file ijms-27-09395-s001.zip › ijms-4624861-supplementary.pdf]

Supplementary Tables

Table S1. Day-stratified Mann–Whitney comparisons of tissue VEGF.

Day	Comparison	Stratum	Group A	A median (min–max), pg/mg protein	Group B	B median (min–max), pg/mg protein	U	Raw p	Holm-adjusted p
3	Healthy vs diabetes	pooled across LLLT status	Healthy	6.54 (2.84–7.88)	Diabetes	3.24 (0.90–5.97)	125	0.002	0.006
3	No LLLT vs LLLT	pooled across metabolic status	No LLLT	2.78 (0.90–6.52)	LLLT	6.26 (3.76–7.88)	17	0.002	0.006
3	No LLLT vs LLLT	Healthy	No LLLT	4.72 (2.84–6.52)	LLLT	7.47 (6.55–7.88)	0	0.002	—
3	No LLLT vs LLLT	Diabetes	No LLLT	1.79 (0.90–2.72)	LLLT	4.75 (3.76–5.97)	0	0.002	—
3	Healthy vs diabetes	No LLLT	Healthy	4.72 (2.84–6.52)	Diabetes	1.79 (0.90–2.72)	36	0.002	—
3	Healthy vs diabetes	LLLT	Healthy	7.47 (6.55–7.88)	Diabetes	4.75 (3.76–5.97)	36	0.002	—
7	Healthy vs diabetes	pooled across LLLT status	Healthy	4.27 (2.53–6.31)	Diabetes	3.08 (0.42–4.53)	119	0.007	0.014
7	No LLLT vs LLLT	pooled across metabolic status	No LLLT	3.19 (0.42–4.89)	LLLT	4.26 (2.15–6.31)	37	0.046	0.092
7	No LLLT vs LLLT	Healthy	No LLLT	3.84 (2.53–4.89)	LLLT	4.50 (3.96–6.31)	9	0.180	—
7	No LLLT vs LLLT	Diabetes	No LLLT	2.17 (0.42–3.71)	LLLT	3.70 (2.15–4.53)	6	0.065	—
7	Healthy vs diabetes	No LLLT	Healthy	3.84 (2.53–4.89)	Diabetes	2.17 (0.42–3.71)	31	0.041	—
7	Healthy vs diabetes	LLLT	Healthy	4.50 (3.96–6.31)	Diabetes	3.70 (2.15–4.53)	30	0.065	—

10	Healthy vs diabetes	pooled across LLLT status	Healthy	1.48 (1.13–1.80)	Diabetes	1.47 (0.56–2.27)	65	0.707	0.707
10	No LLLT vs LLLT	pooled across metabolic status	No LLLT	1.51 (0.56–2.27)	LLLT	1.43 (1.14–1.80)	69	0.885	0.885
10	No LLLT vs LLLT	Healthy	No LLLT	1.51 (1.13–1.60)	LLLT	1.42 (1.26–1.80)	15	0.699	—
10	No LLLT vs LLLT	Diabetes	No LLLT	1.73 (0.56–2.27)	LLLT	1.47 (1.14–1.69)	20	0.818	—
10	Healthy vs diabetes	No LLLT	Healthy	1.51 (1.13–1.60)	Diabetes	1.73 (0.56–2.27)	14	0.589	—
10	Healthy vs diabetes	LLLT	Healthy	1.42 (1.26–1.80)	Diabetes	1.47 (1.14–1.69)	19	0.937	—

Values are tissue VEGF concentrations expressed as pg/mg total protein and are reported as median (minimum–maximum). Comparisons were performed using the Mann–Whitney U test. For the two prespecified pooled contrast families—healthy vs diabetes pooled across LLLT status and no LLLT vs LLLT pooled across metabolic status—p values were adjusted across healing days 3, 7, and 10 using the Holm procedure. Within-stratum comparisons were exploratory and are reported with unadjusted p values only. A dash indicates that no multiplicity-adjusted p value was calculated for the exploratory within-stratum comparison. Abbreviations: LLLT, low-level laser therapy; VEGF, vascular endothelial growth factor.

Table S2. Individual histopathological parameters by experimental group and healing day.

Day	Group	n	Necrosis	Neutrophils	Macrophages	Lymphocytes	Plasma cells	Vascularization	Fibroblasts	Granulation tissue
3	Healthy + ulcer	6	2.0 (0–3)	2.0 (1–3)	1.0 (1–2)	0.0 (0–0)	0.0 (0–0)	1.0 (1–2)	0.0 (0–1)	1.0 (1–2)
3	Healthy + ulcer + LLLT	6	1.5 (0–2)	2.0 (1–3)	1.0 (1–2)	0.0 (0–0)	0.0 (0–0)	2.0 (2–3)	1.0 (0–1)	1.5 (0–3)
3	Diabetes + ulcer	6	2.0 (1–3)	3.0 (2–3)	2.0 (1–3)	0.0 (0–0)	0.0 (0–0)	1.0 (1–2)	0.0 (0–0)	1.0 (0–2)
3	Diabetes + ulcer + LLLT	6	1.0 (0–3)	2.0 (2–3)	1.0 (1–1)	0.0 (0–0)	0.0 (0–0)	2.0 (1–2)	0.0 (0–2)	1.0 (0–2)
7	Healthy + ulcer	6	0.0 (0–0)	0.0 (0–0)	0.0 (0–0)	1.0 (1–2)	1.0 (1–2)	1.0 (1–2)	2.5 (2–3)	0.0 (0–1)
7	Healthy + ulcer + LLLT	6	0.0 (0–0)	0.0 (0–0)	0.0 (0–0)	2.0 (1–3)	2.0 (1–3)	1.0 (1–2)	3.0 (2–3)	0.0 (0–0)
7	Diabetes + ulcer	6	0.0 (0–2)	0.0 (0–2)	0.0 (0–2)	1.0 (0–2)	1.0 (1–2)	1.0 (1–2)	2.0 (1–3)	0.0 (0–2)
7	Diabetes + ulcer + LLLT	6	0.0 (0–0)	0.0 (0–0)	0.0 (0–0)	1.0 (1–2)	1.0 (1–2)	1.0 (1–2)	2.5 (2–3)	0.0 (0–0)
10	Healthy + ulcer	6	0.0 (0–0)	0.0 (0–0)	0.0 (0–0)	1.0 (1–2)	1.0 (0–2)	1.0 (1–1)	3.0 (2–3)	0.0 (0–0)
10	Healthy + ulcer + LLLT	6	0.0 (0–0)	0.0 (0–0)	0.0 (0–0)	1.0 (1–1)	1.0 (1–1)	1.5 (1–2)	3.0 (3–3)	0.0 (0–0)
10	Diabetes + ulcer	6	0.0 (0–0)	0.0 (0–0)	0.0 (0–0)	2.0 (1–3)	2.0 (1–3)	1.0 (0–1)	2.0 (2–3)	0.0 (0–0)
10	Diabetes + ulcer + LLLT	6	0.0 (0–0)	0.0 (0–0)	0.0 (0–0)	1.0 (1–1)	1.0 (1–1)	1.0 (1–2)	3.0 (2–3)	0.0 (0–0)

Values are median (minimum–maximum). Histopathological features were scored on a four-point semiquantitative scale: 0 = absent, 1 = mild, 2 = moderate, and 3 = severe. Each experimental group included n = 6 animals at each healing day. Abbreviation: LLLT, low-level laser therapy.

Table S3A. Kruskal–Wallis analysis of individual and derived histopathological outcomes

Outcome	Day	H	p
Necrosis	3	3.392	0.335
Necrosis	7	3.000	0.392
Necrosis	10	NA	NA
Neutrophils	3	3.711	0.294
Neutrophils	7	6.261	0.100
Neutrophils	10	NA	NA
Macrophages	3	7.130	0.068
Macrophages	7	6.261	0.100
Macrophages	10	NA	NA
Lymphocytes	3	NA	NA
Lymphocytes	7	2.862	0.413
Lymphocytes	10	9.556	0.023
Plasma cells	3	NA	NA
Plasma cells	7	4.850	0.183
Plasma cells	10	7.740	0.052
Vascularization	3	8.199	0.042
Vascularization	7	0.726	0.867
Vascularization	10	7.213	0.065
Fibroblasts	3	6.278	0.099
Fibroblasts	7	3.502	0.320
Fibroblasts	10	5.750	0.124
Granulation tissue	3	1.203	0.752
Granulation tissue	7	4.263	0.234
Granulation tissue	10	NA	NA

Inflammation/injury score	3	8.123	0.044
Inflammation/injury score	7	2.164	0.539
Inflammation/injury score	10	7.740	0.052
Tissue repair score	3	8.462	0.037
Tissue repair score	7	1.407	0.704
Tissue repair score	10	11.857	0.008

Kruskal–Wallis tests compared the four ulcer-bearing experimental groups within each healing day. Histopathological component scores and the two derived composite scores were analyzed as semiquantitative outcomes. NA indicates absence of outcome variability, precluding calculation of the Kruskal–Wallis statistic. These analyses were exploratory and were not adjusted for multiplicity. Binary mucosal continuity was analyzed separately using the Fisher–Freeman–Halton exact test and is summarized in Supplementary Table S3B; day 3 was not tested because there was no outcome variability.

Table S3B. Binary mucosal continuity by experimental group and healing day

Day	Healthy + ulcer	Healthy + ulcer + LLLT	Diabetes + ulcer	Diabetes + ulcer + LLLT	Exact p
3	0/6 (0%)	0/6 (0%)	0/6 (0%)	0/6 (0%)	NA*
7	2/6 (33.3%)	4/6 (66.7%)	1/6 (16.7%)	3/6 (50.0%)	0.524
10	5/6 (83.3%)	6/6 (100%)	4/6 (66.7%)	5/6 (83.3%)	0.878

Values are n/N (%) of animals with restored mucosal continuity. Between-group comparisons within each healing day were performed using the Fisher–Freeman–Halton exact test. *Day 3 was not tested because all animals had absent mucosal continuity and there was no outcome variability. Abbreviation: LLLT, low-level laser therapy.

Table S4. Rank-based multivariable models for individual histopathological outcomes

**Outcome**	**Day 7 vs 3, β (95% CI); p**	**Day 10 vs 3, β (95% CI); p**	**DM, β (95% CI); p**	**LLLT, β (95% CI); p**	**Rank-transformed VEGF, β (95% CI); p**	**R^2**	**n**
Necrosis	−31.139 (−36.694 to −25.583); <0.001	−37.132 (−45.557 to −28.707); <0.001	0.076 (−2.854 to 3.007); 0.959	−1.057 (−5.455 to 3.340); 0.637	−0.172 (−0.403 to 0.059); 0.144	0.763	72
Neutrophils	−34.710 (−39.110 to −30.310); <0.001	−38.864 (−44.732 to −32.995); <0.001	2.165 (−1.063 to 5.394); 0.189	−1.686 (−5.082 to 1.711); 0.331	−0.068 (−0.227 to 0.091); 0.399	0.901	72
Macrophages	−33.266 (−38.561 to −27.971); <0.001	−38.701 (−45.518 to −31.885); <0.001	1.481 (−2.624 to 5.585); 0.480	−2.860 (−6.993 to 1.273); 0.175	−0.087 (−0.277 to 0.102); 0.367	0.861	72
Lymphocytes	37.202 (31.116 to 43.287); <0.001	36.931 (28.460 to 45.403); <0.001	0.357 (−5.172 to 5.886); 0.899	−2.867 (−8.635 to 2.901); 0.330	0.090 (−0.098 to 0.278); 0.349	0.742	72
Plasma cells	38.169 (32.905 to 43.432); <0.001	39.913 (31.959 to 47.866); <0.001	3.174 (−2.544 to 8.891); 0.277	−2.833 (−8.256 to 2.589); 0.306	0.188 (0.013 to 0.363); 0.036	0.765	72
Vascularization	−14.448 (−24.097 to −4.799); 0.003	−18.334 (−33.289 to −3.380); 0.016	−6.305 (−13.907 to 1.298); 0.104	14.095 (5.671 to 22.519); 0.001	−0.112 (−0.474 to 0.250); 0.543	0.311	72
Fibroblasts	32.606 (26.907 to 38.305); <0.001	33.305 (25.040 to 41.570); <0.001	−7.061 (−12.174 to −1.948); 0.007	8.462 (3.447 to 13.477); <0.001	−0.111 (−0.317 to 0.096); 0.294	0.769	72
Granulation tissue	−27.002 (−34.587 to −19.416); <0.001	−34.679 (−43.778 to −25.579); <0.001	−1.170 (−6.902 to 4.562); 0.689	−1.679 (−6.747 to 3.389); 0.516	−0.108 (−0.360 to 0.144); 0.400	0.636	72

Models were fitted as rank(outcome) ~ diabetes status + LLLT treatment + healing day + rank-transformed VEGF, with day 3 as the reference category. β coefficients are reported on the rank-transformed outcome scale with heteroskedasticity-consistent HC3 95% confidence intervals and p values. Positive coefficients indicate higher outcome ranks and should not be interpreted as changes in the original histopathological score units. Component-level analyses were exploratory and were not adjusted for multiplicity. Mucosal continuity was not included because it was recorded as a binary endpoint and analyzed separately. Abbreviations: CI, confidence interval; DM, diabetes mellitus; LLLT, low-level laser therapy; VEGF, vascular endothelial growth factor.

Table S5. Day-3 VEGF–histology associations before and after adjustment for diabetes and LLLT

Histological outcome	Spearman ϱ	Unadjusted p	Adjusted VEGF β (95% CI)	HC3 p	Model R^2	n
Tissue repair score	0.486	0.016	−0.181 (−1.017 to 0.655)	0.671	0.363	24
Vascularization	0.508	0.011	0.178 (−0.848 to 1.205)	0.734	0.321	24
Fibroblasts	0.495	0.014	0.197 (−0.490 to 0.884)	0.574	0.279	24
Inflammation/injury score	−0.521	0.009	−0.138 (−1.395 to 1.119)	0.830	0.312	24

Unadjusted associations are Spearman rank correlations. Adjusted models were restricted to day 3 and were fitted as rank(histological outcome) ~ rank-transformed VEGF + diabetes status + LLLT treatment. β coefficients for rank-transformed VEGF are reported on the rank-transformed outcome scale with heteroskedasticity-consistent HC3 95% confidence intervals and p values. None of the VEGF terms remained statistically significant after adjustment for diabetes status and LLLT treatment. Abbreviations: CI, confidence interval; DM, diabetes mellitus; LLLT, low-level laser therapy; VEGF, vascular endothelial growth factor.

Table S6. Construction of exploratory histopathological composite scores

Score	Equal-weight components	Each component	Total score range
**Inflammation/injury**	Necrosis + neutrophils + macrophages + lymphocytes + plasma cells	0–3	0–15
**Tissue repair**	Vascularization + fibroblasts + granulation tissue	0–3	0–9

Each component was the mean of two blinded pathologists' independent four-point ratings (0 = absent, 1 = mild, 2 = moderate, 3 = severe). Component means were summed with equal weights for each animal. The two scores are exploratory, study-specific summary indices and have not been formally validated. Mucosal continuity was assessed separately as a binary endpoint and was not included in either composite score.

Table S7. Post hoc leave-one-component-out sensitivity analyses of the LLLT association with exploratory tissue repair.

Score definition	LLLT β (rank scale)	HC3 95% CI lower	HC3 95% CI upper	Unadjusted p
Full composite	17.489	8.541	26.436	0.00013
Without vascularization	10.192	2.838	17.546	0.00660
Without fibroblasts	7.875	0.438	15.312	0.03795
Without granulation tissue	15.380	8.604	22.157	0.00001

Models: rank(score) ~ diabetes + LLLT + categorical healing day + rank(VEGF), using HC3 robust covariance. These were post hoc exploratory checks without multiplicity correction. Coefficients are rank-scale estimates and do not quantify changes in the original score units.

Supplementary Figures

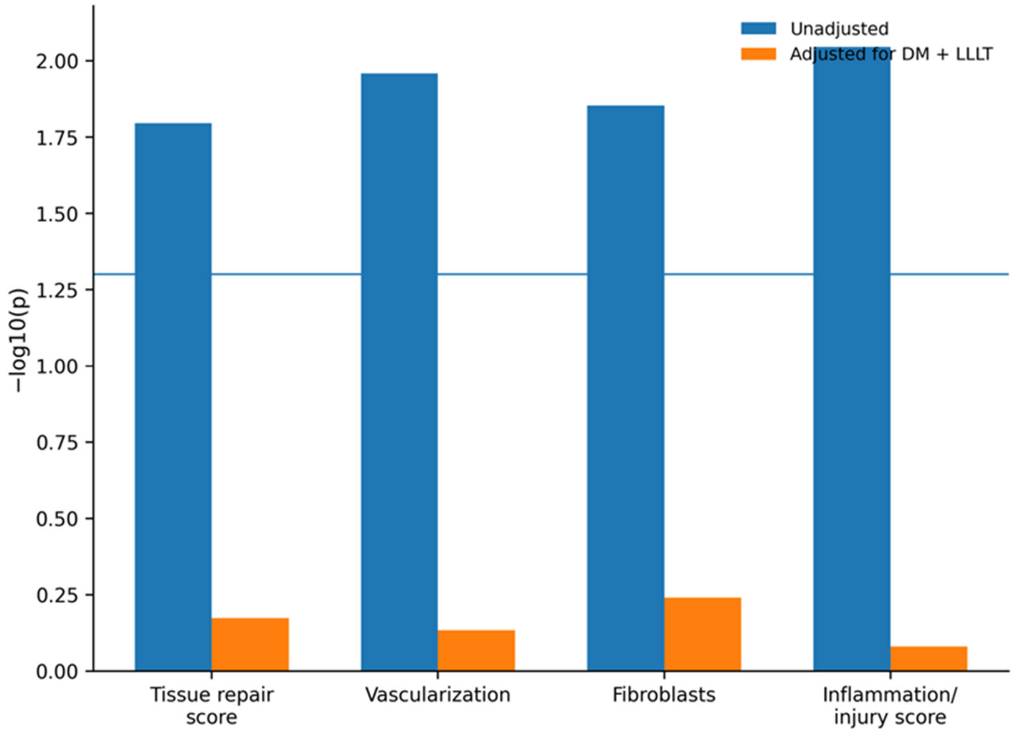

Supplementary Figure S1. Day-3 VEGF–histology associations before and after adjustment for diabetes and LLLT.

Bars represent −log10(p) values for unadjusted Spearman associations and corresponding adjusted VEGF terms from rank-based models including diabetes status and LLLT treatment. The horizontal reference line corresponds to p = 0.05. None of the VEGF terms remained statistically significant after adjustment. Abbreviations: DM, diabetes mellitus; LLLT, low-level laser therapy; VEGF, vascular endothelial growth factor.

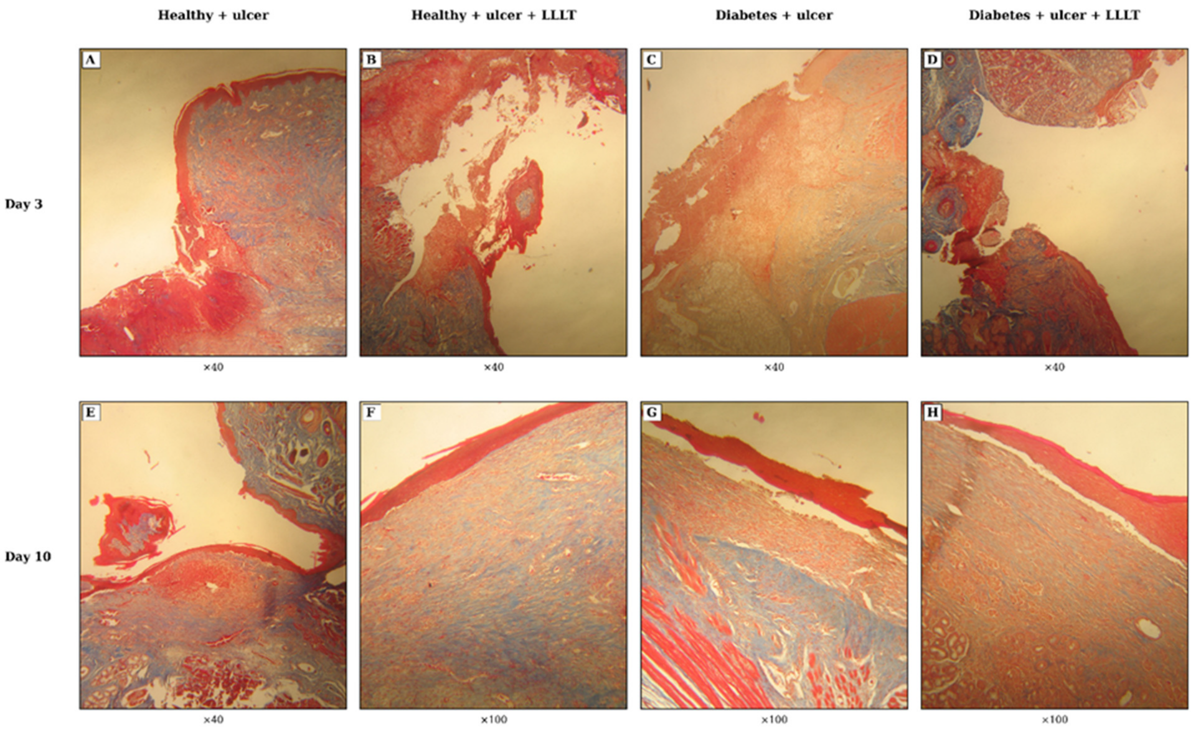

Supplementary Figure S2. Representative Masson trichrome-stained sections across experimental groups and healing phases.

(A–D) Day 3: healthy + ulcer (A; ×40), healthy + ulcer + LLLT (B; ×40), diabetes + ulcer (C; ×40), and diabetes + ulcer + LLLT (D; ×40). (E–H) Day 10: healthy + ulcer (E; ×40), healthy + ulcer + LLLT (F; ×100), diabetes + ulcer (G; ×100), and diabetes + ulcer + LLLT (H; ×100). Panels are representative examples from the archived histopathological documentation and are provided as complementary morphological illustrations. Representative cropped views from the original histopathological micrographs are shown without local image enhancement; corresponding uncropped source images are retained and available if required. Abbreviation: LLLT, low-level laser therapy.

Supplementary Figure S3. Post hoc sensitivity of the adjusted LLLT–tissue repair association.

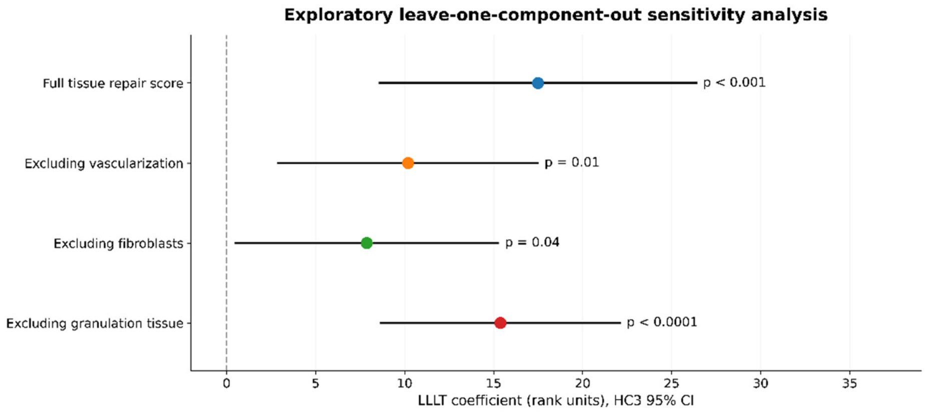

Markers show adjusted LLLT coefficients, error bars show HC3 95% confidence intervals. Every reduced composite is ranked separately; numerical coefficients across different outcomes are not directly comparable as effect magnitudes. Exploratory analyses were not multiplicity-adjusted. LLLT, low-level laser therapy; VEGF, vascular endothelial growth factor.
